# Knowledge, attitude, and practice of hospice care among senior nursing staff in Chongqing and Hebei: a cross-regional study

**DOI:** 10.3389/fpubh.2025.1512897

**Published:** 2025-05-21

**Authors:** Huan Liu, Ying Zeng, Qin Chen, Jihua Feng, Lu Wang

**Affiliations:** ^1^Department of Geriatric, Chongqing General Hospital, Chongqing University, Chongqing, China; ^2^Department of Geriatric, TongNan Traditional Chinese Medicine Hospital, Chongqing, China; ^3^Department of Geriatric, HanDan Central Hospital, Hebei, China; ^4^Department of Oncology, Chongqing General Hospital, Chongqing University, Chongqing, China

**Keywords:** hospice care, gerontological advanced practice nurses (GAPNs), knowledge, attitude, practice, China

## Abstract

**Background:**

The demand for quality hospice care rises in China as the population ages. Specialized gerontological advanced practice nurses (GAPNs) are vital to the Chinese healthcare system, yet their knowledge, attitudes, and practices (KAP) regarding hospice care in China remain poorly understood.

**Objectives:**

To explore the KAP toward hospice care in specialized GAPNs and the factors influencing KAP.

**Methods:**

This cross-sectional study was conducted from May 6, 2023, to July 30, 2023, in GAPNs working in Chongqing and Hebei. This study used a previously developed scale on the KAP toward hospice care. Descriptive analysis, univariable and multivariable regression analyses, and correlation analyses were used.

**Results:**

A total of 300 valid questionnaires were collected. The median knowledge score was 7. The median attitude score was 82. The proportion of participants with hospice care experience was 15.7%. The median confidence in practice score was 38. The median actual practice score was 40. High school/vocational school education and unwillingness to participate in hospice care were negatively associated with knowledge. Unwillingness to participate in hospice care and knowledge scores were positively associated with attitudes. Age 41–50 and attitude scores were positively associated with confidence in practice, while never provided hospice care and Hebei province were negatively associated.

**Conclusion:**

GAPNs in Chongqing and Hebei show moderate KAP toward hospice care, with Chongqing performing better. The regional differences found suggest the need for region-specific education and policy interventions.

## Introduction

With the continuous advancement of modern medicine and improved living conditions, the average life expectancy has significantly increased worldwide (1), but it also ushered in the acceleration of population aging (2). According to the Seventh National Population Census of China and relevant statistics from the National Bureau of Statistics, by the end of 2021, the population of individuals aged >60 in China reached 267.36 million (18.9% of the total population), while the population of individuals aged >65 in China amounted to 200.56 million (14.2% of the total population) (3). It signifies that China has officially entered a “profoundly aging society,” with the standard for such a society being 20% of the population aged >60 and >14% of the population aged >65 (4, 5). Furthermore, it is anticipated that the degree of aging will continue to deepen.

Therefore, ensuring a comfortable, dignified, and peaceful living and departure for China’s vast aging population has become a critical aspect of the entire life cycle and the holistic health process (6–8). Hospice care (HC) is a comprehensive model of healthcare that integrates medical services, life care, and psychological support (7, 9). It is designed to alleviate pain and discomfort in patients or older adults with a life expectancy of <6 months, offering emotional comfort, addressing their social and spiritual needs, and providing emotional support and grief counseling to their family members. HC ensures that patients or older adults depart this world with dignity and without regrets (6–8). Notably, HC significantly reduces the costs of caring for late-stage patients or older adults and yields substantial economic benefits.

In response to the progressively deepening aging of the population and the increasing demand for quality end-of-life care, the Chinese government promulgated the “Guidelines for Hospice Care Practices (Pilot)” in 2017 (10), which aimed to guide various institutions in developing HC. Simultaneously, China has taken several measures to establish institutional and community care infrastructure as alternatives and supplements to home-based care (11, 12). Despite the government’s prioritization of HC in its policy agenda and the substantial progress made in HC services in China, as of now, the discipline of HC, which focuses on symptom control, improving the quality of life for patients, enhancing the quality of end-of-life care, and assisting patients’ family members and caregivers, is still in its early stages (6, 7). The system is not yet fully developed, public awareness is relatively low, and healthcare professionals are scarce. These factors result in the limited availability and quality of HC in China (12).

Doctors and nurses play pivotal roles in HC teams, with nurses serving as the primary healthcare providers and most trusted individuals for end-of-life patients and their families, and are integral throughout the entire HC process (13). Gerontological advanced practice nurses (GAPNs) are specialized healthcare professionals delivering older adult care and are the most suitable candidates for meeting the aging population’s needs and providing HC (14). Their knowledge, attitudes, and practices (KAP) significantly influence the quality and development of the HC industry in China.

The provision of HC during emergencies and disasters is challenging, as was observed during the recent COVID-19 pandemic. By definition, disasters are events that lead to the massive disruption of the proper functioning of a community or society due to events that exceed the community’s or society’s capacity to cope with the event (e.g., biological, geophysical, meteorological, hydrological, wars, etc.) (15, 16). In disasters, vulnerable populations are particularly of poor outcomes due to the disruption of services essential to their welfare (17, 18). Hence, because of limited autonomy and reliance on others, people receiving HC are particularly vulnerable to disasters (19). The delivery of HC during disasters can be difficult because resources are directed toward controlling the disaster and treating the victims, and triage can even be implemented (20, 21). In addition, differences in medical resources and cultural backgrounds can influence the provision of HC in different parts of large countries such as China (22–24). Indeed, due to the vast size of China, there are significant differences in economy, financial, human, and medical resources, cultural backgrounds, and policy implementation among different Chinese Provinces. Therefore, although there have been some studies on HC among healthcare providers in China (22–24), it is necessary to conduct in-depth research on the different regions and Provinces in the country. It is why this study focused on the KAP of GAPNs in the Chongqing and Hebei regions of China.

It was hypothesized that the KAP of GAPNs is significantly correlated with the medical resources and educational background of the region in which they are located. Therefore, this cross-sectional study assessed the KAP toward HC in specialized GAPNs in multiple hospitals in Chongqing and Hebei, China, and explored the factors influencing KAP. This research will serve as a basis for optimizing HC training programs in Chongqing and Hebei, contributing to the advancement of high-quality HC services and holding significant practical significance. This study will also fill a research gap in this specific population and region. In addition, the Chongqing and Hebei regions because of their socioeconomic disparities.

## Methods

### Study design and participants

This cross-sectional study was conducted from May 6, 2023, to July 30, 2023, in Chongqing General Hospital and in HanDan Central Hospital. The study focused on GAPNs. Ethical approval for this study was obtained from the Medical Ethics Committee of Chongqing General Hospital. Written informed consent was obtained from all participants online.

In this study, the survey population was selected using the convenience sampling method. The inclusion criteria were (1) aged >18 years, (2) possessed relevant nursing qualifications and had a minimum of 5 years of work experience, and (3) the ability to independently complete the questionnaire.

### Sample size determination

The sample size was calculated using the following formula:


$$n=\frac{z^{2}p(1-p)}{d^{2}}$$

where z = 1.96 at a 5% level of significance and a 5% acceptable margin of error (d = 0.05). The proportion of the expected population based on previous studies or pilot studies was set at 9% to maximize the sample size. Based on the above, the sample size was calculated as 126 (25).

### Questionnaires and data collection

This study used the KAP toward HC scale developed by Shu et al. (24), demonstrating good reliability and validity. A small-scale pilot test (31 questionnaires) yielded a Cronbach’s *α* of 0.943.

The scale consisted of four parts: demographic characteristics (11 items), knowledge (15 items), attitudes (24 items with five sub-concepts), and practices (two sub-concepts: confidence in practices and self-reported practices, each containing 11 items) related to HC. Only GAPNs with HC experience were requested to respond to items regarding self-reported practices. The scores for each subcategory were calculated separately. For the knowledge section, responses were scored as 1 for “true” and 0 for “false” or “unsure.” The attitude section was assessed using a five-point Likert scale, ranging from “totally agree” (5) to “totally disagree” (1). Scores for negative topics (items A1 and A4) were reverse-coded before calculation. The other two sections (confidence and self-reported practices) were also measured using a five-point Likert scale, with options ranging from “rather confident” (5) to “rather non-confident” (1) for confidence and “always” (5) to “never” (1) for self-reported practices. KAP scores ≥80% were considered good knowledge, positive attitudes, and proactive practice (26).

An online questionnaire with a QR code was created using the WeChat-based Questionnaire Star applet for data collection via WeChat. The questionnaire was distributed to the WeChat group of the 2022 National Geriatric Talent Nurse Training Course in Chongqing and the WeChat group of geriatric nurses at *** Hospital. The participants accessed the questionnaire by scanning the QR code and completing it through the WeChat platform. The entry page was the informed consent form; signing it was mandatory to gain access to the questionnaire itself. All questionnaires were answered anonymously.

The questionnaire contained a question as to whether the participant had any experience in HC. The number of questions was different according to HC experience. The quality control exclusion criteria were (1) took <80 s (when indicating no hospice care experience), <100 s (when indicating hospice care experience), or >1800 s to complete the questionnaire, (2) incomplete questionnaire, or (3) questionnaires with inconsistent responses to items. Quality control was done by a third party. Participation was not mandatory. The participants had to understand the items by themselves without exterior help, which also represents their level of understanding of hospice care. The questionnaire has been validated for use by healthcare providers, and its language and content should not be problematic (24).

### Data analysis

The reliability and validity of the questionnaire were evaluated using the Kaiser-Meyer-Olkin (KMO) and Cronbach’s *α* methods (27). The data were analyzed using SPSS 22.0 (IBM, Armonk, NY, United States). The demographic characteristics and dimension scores were analyzed descriptively. The Kolmogorov–Smirnov test was used to determine the distribution of the KAP scores. If the data met the criteria for a normal distribution, the data were presented as means ± standard deviations and analyzed using Student’s t-test (comparison of two groups) or ANOVA (comparison of more than two groups); otherwise, they were presented as medians (interquartile ranges (IQRs)) and analyzed using the Mann–Whitney U-test (comparison of two groups) or the Kruskal-Wallis H-test (comparison of more than two groups). The categorical data were presented as *n* (%) and analyzed using the chi-squared test. Correlations between KAP dimensions were analyzed using Pearson correlation analysis (normal distribution) or Spearman correlation analysis (non-normal distribution), with *p*-value correction using the Bonferroni correction. Univariable and multivariable regression analyses were performed using the KAP scores as the dependent variables. Continuous variables were dichotomized based on their mean (normal distribution) or median (non-normal distribution). The variables with *p* < 0.10 in the univariable analyses were included in the multivariable analyses. The *p*-values were reported with three decimals. Two-sided *p*-values <0.05 were considered statistically significant.

## Results

### Characteristics of the participants

A total of 414 questionnaires were collected, of which 300 (72.5%) were valid, including 159 from Chongqing and 141 from Hebei. Most invalid questionnaires were excluded because they were incomplete. The questionnaires showed good reliability and validity, with Cronbach’s *α* of 0.895 and a Kaiser-Meyer-Olkin (KMO) of 0.906. The Cronbach’s α for knowledge, attitudes, confidence, and practices were 0.779, 0.750, and 0.965, respectively. The scores for knowledge and confidence in practice did not conform to a normal distribution. The attitude and actual practice scores followed a normal distribution (Table S1). For uniformity of the results, all results were presented using medians (IQRs).

The basic characteristics of the participants are presented in Table S2. The results indicate that most participants were female (88.7%), 84.6% were below 40, and 65.7% held a bachelor’s degree or higher. Nearly half (56.3%) held junior or below job titles, while three-quarters (75.7%) had personal experience with the death of terminally ill patients or relatives. Regarding hospice care service practice, only 15.7% of GAPNs had experience, but 75.7% of GAPNs were willing to participate in HC services. Among the reasons participants were willing to provide HC services, personal duty accounted for 54.2%, followed by tasks assigned by superiors at 37%. Conversely, the most common reason participants were unwilling to provide HC was the high level of stress (63%).

A comparison of the basic characteristics of GAPNs in Chongqing and Hebei is presented in Table S3. The results showed that there were more male participants in Chongqing than in Hebei. Compared to Chongqing, more GAPNs in Hebei possessed a bachelor’s degree or higher education and held junior titles or higher. Other remaining basic characteristics of GAPNs in the two regions were similar.

### Knowledge

The median knowledge score was 7 (/15, 46.67%; IQR: 4–9; range: 0–12; 59.7% with scores >median) (Table 1). The scores were similar between Chongqing and Hebei (Table 1). Higher knowledge scores were observed in GAPNs of 41–50 years (*p* < 0.001), with higher education (*p* < 0.001), higher professional title (*p* < 0.001), with experience with terminal illness (*p* < 0.001), with HC experience (*p* = 0.011), and willing to provide HC (*p* < 0.001) (Table S2). The item with the highest score was K2 (79.7%; “Psychological, social, and spiritual issues are crucial for an HC team to provide appropriate counseling and management,”) while the item with the lowest score was K8 (3.7%; “Applying potassium permanganate at the Shenque acupoint can alleviate ascites symptoms”) (Table S4).

**Table 1:** KAP scores.

KAP score distribution	Possible range	Total score median	P25	P75	Minimum	Maximum	<Median, *n* (%)	≥Median, *n* (%)
Overall (*n* = 300)								
Knowledge	0 ~ 10	7	4	9	0	12	121 (40.3)	179 (59.7)
Attitude	63 ~ 118	82	77	90	57	118	146 (48.7)	154 (51.3)
Confidence in practice	24 ~ 55	38	33	44.75	11	55	145 (48.3)	155 (51.7)
Actual practice (*n* = 47)	11 ~ 55	40	31	46	11	55	21 (44.7)	26 (55.3)
Chongqing (*n* = 159)								
Knowledge	0 ~ 10	8	4	9	0	11	58 (36.5)	101 (63.5)
Attitude	63 ~ 118	82	75	90	57	118	78 (49.1)	81 (50.9)
Confidence in practice	24 ~ 55	40	33	45	18	55	67 (42.1)	92 (57.9)
Actual practice (*n* = 28)	11 ~ 55	42	36.5	48.5	22	55	10 (35.7)	18 (64.3)
Hebei (*n* = 141)								
Knowledge	0 ~ 10	7	4	9	0	12	63 (44.7)	78 (55.3)
Attitude	63 ~ 118	82	78	90	63	115	68 (48.2)	73 (51.8)
Confidence in practice	24 ~ 55	35	33	44	11	55	78 (55.3)	63 (44.7)
Actual practice (*n* = 19)	11 ~ 55	34	27	43	11	55	11 (57.9)	8 (42.1)

### Attitudes

The median attitude score was 82 (/120, 68.33%; IQR: 77–90; range: 57–118; 51.3% with scores >median) (Table 1). The scores were similar between Chongqing and Hebei (Table 1). Higher attitude scores were observed in females (*p* = 0.001), in the 41–50 age group (*p* = 0.004), with higher education (*p* < 0.001), with higher professional title (*p* < 0.001), with experience with terminal illness (*p* = 0.014), willing to provide HC (*p* < 0.001), and willing to provide HC for personal duty (*p* < 0.001) (Table S2). The items with the highest scores were A9 and A10 (81.4%; “Offer emotional support.” and “Receive support from the family”), while the item with the lowest score was A19 (18.0%; “Late-stage patients having many challenging symptoms”) (Table S5).

### Practices

The practice section consisted of two sub-concepts: confidence in practices and self-reported practices, each containing 11 items. The proportion of individuals with HC experience was 15.7%.

The median confidence in practice score was 38 (/55, 69.09%; IQR: 33–44.75; range: 11–55; 51.7% with scores >median) (Table 1). Higher scores were observed for GAPNs aged 41–50 (*p* < 0.001), divorced or widowed (*p* < 0.001), with higher education (*p* < 0.001), in Chongqing (*p* = 0.030), with higher professional title (*p* < 0.001), with experience in terminal illness (*p* = 0.007), provided HC (*p* < 0.001), willing to provide HC (*p* = 0.009), and willing to provide HC for religious beliefs (*p* < 0.001) (Table S2). The median actual practice score was 40 (/55, 72.73%; IQR: 31–46; range: 11–55; 55.3% with scores >median) (Table 1). Higher scores were observed for GAPNs believing in Buddhism (*p* = 0.043) and in Chongqing (*p* = 0.014) (Table S2). Table S6 shows the distribution of the practice scores, while Tables S7 and S8 show the distribution of the practice scores in Chongqing and Hebei, respectively.

### Correlations

As shown in Table 2, among all GAPNs, the knowledge scores were correlated to the attitude (r = 0.343, *p* < 0.001) and confidence in practice (r = 0.366, *p* < 0.001) scores, while the attitude scores were correlated to the confidence in practice scores (r = 0.519, *p* < 0.001). The threshold after the Bonferroni correction was 0.05/3 = 0.0167. All *p*-values in the first part of Table 2 were <0.001 and are still significant after correction. Among the GAPNs with HC experience, the knowledge scores correlated to the attitude (r = 0.423, *p* = 0.003), confidence in practice (r = 0.567, *p* < 0.001), and actual practice (r = 0.324, *p* = 0.026) scores, the attitude scores correlated to the confidence in practice (r = 0.536, *p* < 0.001) and actual practice (r = 0.374, *p* = 0.010) scores and the confidence in practice scores correlated to the actual practice scores (r = 0.343, *p* = 0.018). There were four variables (including Actual practice; *n* = 47), hence six tests. The Bonferroni threshold was 0.05/6 = 0.0083. Actual practice shows no significant correlation with knowledge, attitudes, and confidence in practice after correctio. Similar patterns were observed in Chongqing (Table S9) and Hebei (Table S10).

**Table 2:** Correlation analysis in all participants.

n = 300	Knowledge	Attitude	Confidence in practice
Knowledge	1.000	/	/
Attitude	0.343 (*P* < 0.001)	1.000	/
Confidence in practice	0.366 (*P* < 0.001)	0.519 (*p* < 0.001)	1.000

*n* = 47	Knowledge	Attitude	Confidence in practice	Actual practice
Knowledge	1.000	/	/	/
Attitude	0.423 (*P* = 0.003)	1.000	/	/
Confidence in practice	0.567 (*P* < 0.001)	0.536 (*P* < 0.001)	1.000	/
Actual practice	0.324 (*P* = 0.026)	0.374 (*P* = 0.010)	0.343 (*P* = 0.018)	1.000

For the first part, the significance threshold after Bonferroni correction was 0.05/3 = 0.0167. For the second part, the significance threshold after Bonferroni correction was 0.05/6 = 0.0083.

### Multivariable analyses

**Figure 1:**
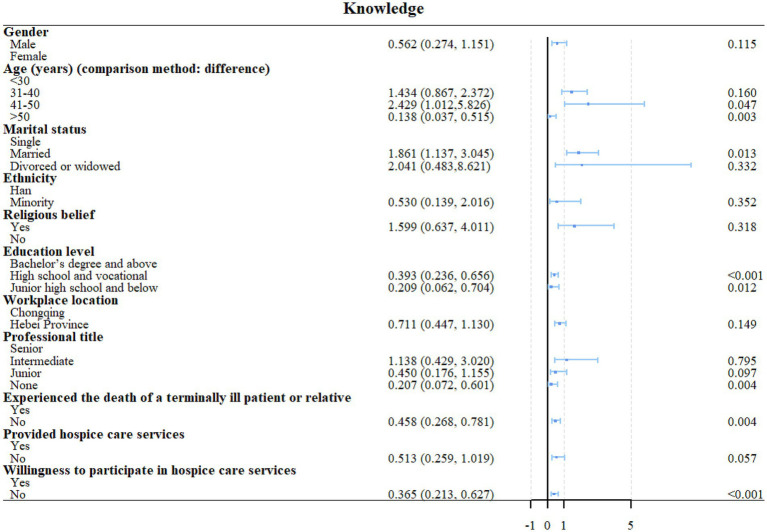
Univariable regression analyses of knowledge.

**Figure 2:**
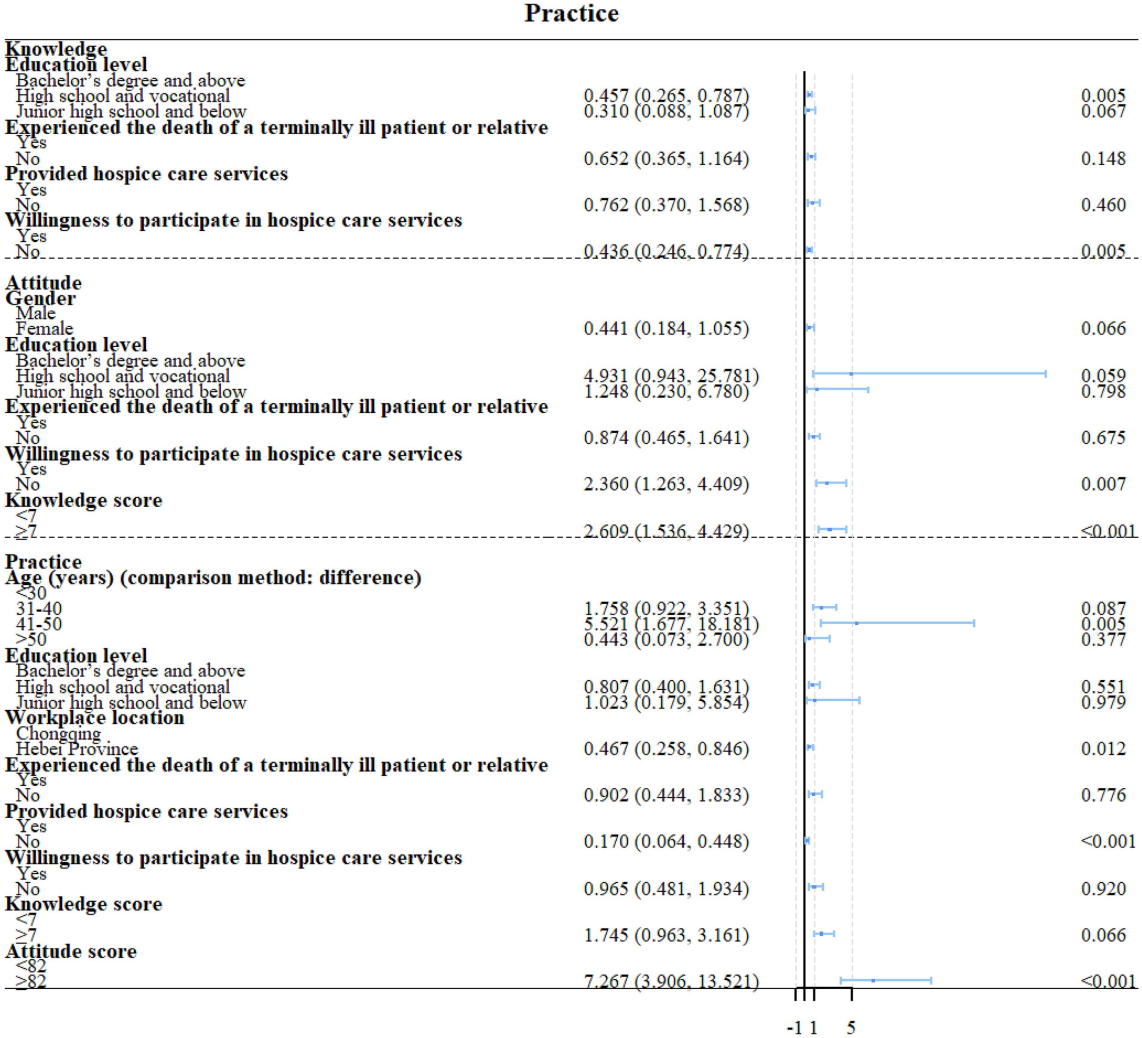
multivariable regression analyses of KAP.

**Figure 3:**
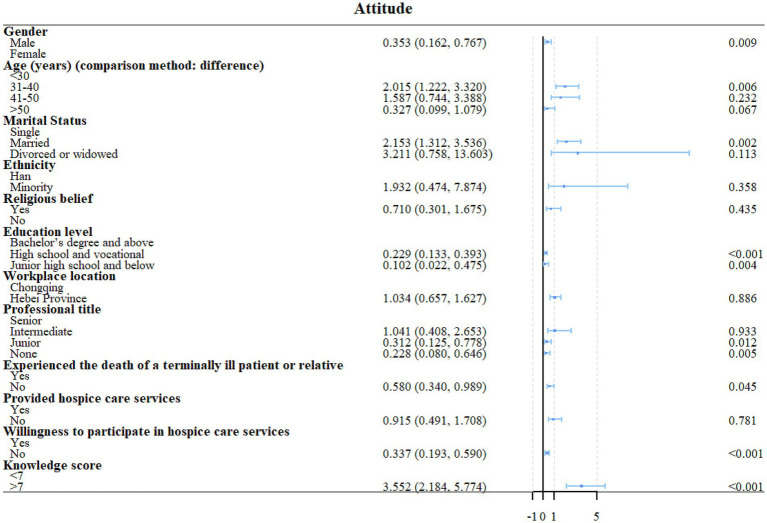
Univariable regression analyses of attitude.

**Figure 4:**
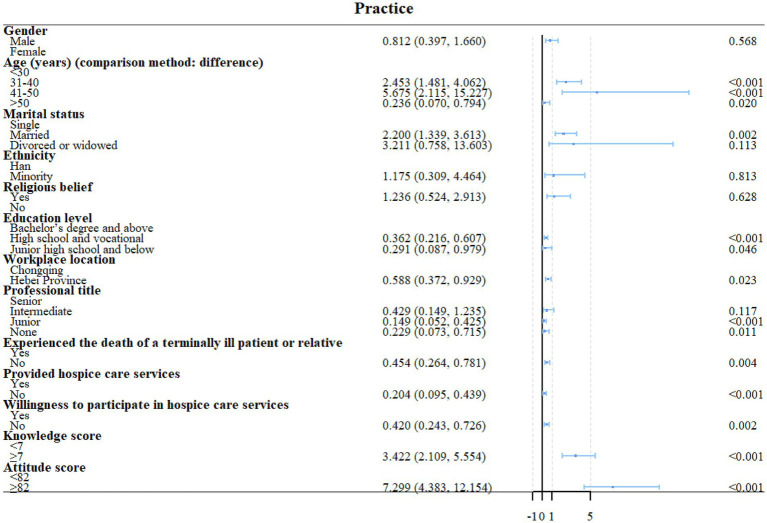
Univariable regression analyses of practice.

High school or vocational school education (OR = 0.457, 95%CI: 0.265–0.787, *p* = 0.005) and unwilling to participate in HC (OR = 0.436, 95%CI: 0.246–0.774, *p* = 0.005) were independently associated with the knowledge scores (Figures 1, 2). Unwillingness to participate in HC (OR = 2.360, 95%CI: 1.263–4.409, *p* = 0.007) and knowledge scores (OR = 2.609, 95%CI: 1.5436–4.429, *p* < 0.001) were independently associated with the attitude scores (Figures 2, 3). Age 41–50 (OR = 5.521, 95%CI: 1.677–18.181, *p* = 0.005), Hebei province (OR = 0.467, 95%CI: 0.258–0.846, *p* = 0.012), never provided HC (OR = 0.170, 95%CI: 0.064–0.448, *p* < 0.001), and attitude scores (OR = 7.267, 95%CI: 3.906–13.521, *p* < 0.001) were independently associated with the confidence in practice scores (Figures 2, 4). In Chongqing, never provided HC (OR = 0.266, 95%CI: 0.079–0.890, *p* = 0.032) and the attitude scores (OR = 9.305, 95%CI: 3.850–22.488, *p* < 0.001) were independently associated with the confidence in practice scores (Table S11). In Hebei, married (OR = 2.773, 95%CI: 1.053–7.306, *p* = 0.039), never provided HC (OR = 0.132, 95%CI: 0.034–0.520, *p* = 0.004), and the attitude scores (OR = 5.401, 95%CI: 2.269–12.857, *p* < 0.001) were independently associated with the confidence in practice scores (Table S12).

## Discussion

This cross-sectional study assessed the KAP toward HC in specialized GAPNs in multiple hospitals in Chongqing and Hebei, China, and explored the factors influencing KAP. GAPNs in Chongqing and Hebei display moderate KAP toward HC. The practice scores are higher in Chongqing than in Hebei. Although this study compared the Chongqing and Hebei regions, it aimed to examine the disparities between the two regions with different socioeconomic statuses rather than compare the nurses in those two regions.

HC is a recent response to China’s issue of the aging population (10), but despite efforts, HC services remain in their early stages (6, 7). GAPNs are highly susceptible to being involved in HC due to their specific skills with older adults (14). Still, there are limited data available regarding their KAP toward HC. The present study reported generally moderate KAP toward HC among GAPNs. These results are similar to the data available from various populations of nurses and other healthcare providers (22, 28, 29), including oncology nurses (30, 31). Poor KAP was also reported for pediatric palliative care (32).

This KAP study revealed the KAP status of GAPNs toward HC in Chongqing and Hebei. Intermediate knowledge level means that the GAPNs have some knowledge of the basic theory and practice of HC but may lack an in-depth and comprehensive understanding. For example, they may know the basic concepts and importance of HC but have limited knowledge of specific care methods, the latest nursing techniques, and relevant laws and regulations. It shows that at the knowledge level, there is room for improvement, which needs to be strengthened through further education and training. The moderate level of attitudes suggests that GAPNs’ attitudes towards HC were generally positive, but this positivity may not be strong or widespread enough. For example, they may recognize the value of HC but may find it difficult to fully support and implement HC in practice due to lack of confidence, insufficient resources, or other work pressures. This suggests that, at the attitudinal level, they need to be motivated through incentives, values education, and improved working environments. Finally, an intermediate level of practice means that the GAPNs demonstrated some competence and skill in actually providing HC but have not yet reached a high level of professional standards. For example, they can provide basic HC and psychological support but may fall short in complex case management, multidisciplinary collaboration, and individualized care planning. It shows that at the practical level, there is a need to improve their practical skills through skills training, experience sharing, and professional guidance.

The present study showed that knowledge, attitudes, and practices were all positively correlated to each other, indicating that improving knowledge should also translate into better attitudes and practices. According to the KAP theory, knowledge is the basis for practice, but attitude is the force driving practice (26, 33). The present study showed that a willingness to participate in HC and actual HC experience were positively associated with the KAP scores. Basic motivation is indeed essential to perform specific tasks in specific populations.

The present study identified several points that should be targeted by continuous education activities or be incorporated into the curriculum of courses about HC. Age 41–50 was independently associated with higher practice scores. The nurses aged 41–50 were the most active, probably because these nurses were experienced yet far enough from retirement to slow down the pace. A lower education was independently associated with lower knowledge scores. Education, in general, is a determinant of healthcare literacy (34). In addition, a lower education in nurses entails less specialized formation. Finally, nurses from Hebei had significantly lower practice scores than their counterparts in Chongqing. Such differences could be related to the differences in the formation curriculums and continuing education resources.

The present study revealed differences in practice scores between Chongqing and Hebei. The two areas display some differences in socioeconomic status, their education system, and their HC resources (35). Chongqing has a population of 32,054,159 (390/km^2^) and a per-capita gross domestic product (GDP) of US$13,479 (36, 37). The government invested in Chongqing to transform the municipality into the economic, trade, and financial center of Western interior China. Hence, Chongqing is facing rapid urbanization. No such special government effort was made for Hebei since the province surrounds Beijing, which is already the economic center of the area. Hebei has a population of 74,610,235 (400/km^2^) and a per-capita GDP of US$8397 (36, 37). In Hebei, 40% of the population works in the agriculture, forestry, and animal husbandry sectors. Hence, HC providers in Hebei displayed lower practice scores, probably related to the lower economic status of Hebei compared with Chongqing, leading to smaller public investments in HC and a smaller capacity of the individuals or their families to pay for HC. Otherwise, the general policies of the Chinese Ministry of Health apply equally in both provinces, and the same practice guidelines are used for HC. These results indicate that the KAP status toward HC should be examined across China to determine the exact needs in education. A strength of the present study is using an externally validated questionnaire (24) that could be used all over China and provide comparable results. Nevertheless, future studies will be performed in areas with more important income disparities. In fact, the exact HC KAP situation of every province and major city in China should be investigated to formulate nationwide recommendations and action plans to optimize HC services across China.

The present study has implications for clinical practice. Indeed, professional knowledge training should be strengthened by regularly organizing special training events on HC in hospitals in Chongqing and Hebei, inviting experts to give lectures and practical guidance, and updating GAPNs’ understanding of the latest nursing methods, technologies, policies, and regulations. E-learning courses could be developed for HC so that the GAPNs can learn and review them anytime, anywhere, ensuring they can continue to develop their expertise. The regional differences should also be addressed through tailored training programs based on the specific conditions of Chongqing and Hebei. For example, in view of the low level of practice in Hebei, priority could be given to increasing the number and intensity of training in the region while providing more resource support. Policy support and resource allocation: It is recommended that the government and relevant departments increase the investment of funds and resources in HC in Hebei Province, improve the level of local medical facilities and equipment, and ensure that GAPNs can receive adequate support in their work. Finally, a multidisciplinary team should be established within the hospital, including doctors, nurses, psychologists, social workers, etc., to participate in HC and provide comprehensive patient care and support.

KAP results are usually highly specific to the area where they were conducted because of the differences in economy, customs, culture, lifestyle habits, access to healthcare, etc., among different regions of the world. The KAP questionnaire used in this study was validated for use in China (24), limiting the direct generalizability of the results to other countries. Still, the study could provide some clues and research directions for countries and areas sharing some similar characteristics. With the authors’ permission (24), the questionnaire could also be adapted and validated to the reality of other countries and areas.

This study had limitations. Even though the study was performed at multiple centers, the sample size remained relatively small. Convenience sampling may limit generalizability. The participants were all recruited from the “2022 National Geriatric Talent Nurse Training Course” in Chongqing and the WeChat groups of geriatric nurses of Chongqing General Hospital and HanDan Central Hospital. Convenience sampling may limit generalizability. The cross-sectional design prevents causality from being examined, and only associations can be reported. Longitudinal studies are necessary to determine causality. In addition, the present study could serve as a baseline to examine the impact of future education interventions or to observe the KAP changes in time. All KAP studies are at risk of the social desirability bias, in which some participants could answer what is socially expected instead of what they are doing (38, 39).

## Conclusion

GAPNs in Chongqing and Hebei display moderate KAP toward HC. The practice scores are higher in Chongqing than in Hebei. The KAP dimension scores are all positively correlated to each other. The willingness to participate in HC is a major determinant in the practice scores toward HC. The results should be used to improve the training and continuing education activities of GAPNs, which could translate into improvements in the quality of HC and the quality of life of the patients.

## Data Availability

The original contributions presented in the study are included in the article/Supplementary material, further inquiries can be directed to the corresponding author.
