# Supplementary material for: Knowledge, attitude, and practice of hospice care among senior nursing staff in Chongqing and Hebei: a cross-regional study
Source: Front Public Health. 2025 May 21;13:1512897. doi: 10.3389/fpubh.2025.1512897 (PMC12133508; doi:10.3389/fpubh.2025.1512897)
Supplement: Supplementary file 1 [file Table_1.doc]

**Table S1. Normality test of the KAP scores**

|  | Kolmogorov-Smirnov normality test |
| --- | --- |
| Knowledge | P<0.001 |
| Attitude | P=0.431 |
| Confidence in practice | P<0.05 |
| Actual practice | P=0.212 |

The scores for knowledge and confidence in practice did not conform to a normal distribution. The attitude and actual practice scores followed a normal distribution. For uniformity of the results, all results were presented using the median (interquartile range).

**Table S2.** Characteristics of the participants (n=300)

| Variables | n (%) | Knowledge | | Attitude | | Practice | | | |
| --- | --- | --- | --- | --- | --- | --- | --- | --- | --- |
|  |  |  |  |  |  | Confidence in practice | | Actual practice score | |
|  |  | Median (IQR) | P | Median (IQR) | P | Median (IQR) | P | Median (IQR) | P |
| Total | 300 | 7 (4, 9) |  | 82 (77, 90) |  | 38 (33, 44.75) |  | 40 (31, 46) |  |
| Gender |  |  | 0.141 |  | 0.001 |  | 0.736 |  | 0.966 |
| Male | 34 (11.3) | 6 (1, 9) |  | 78 (72, 83.25) |  | 36.5 (33, 44.75) |  | 39 (29.75, 49.75) |  |
| Female | 266 (88.7) | 7.5 (4, 9) |  | 82 (78, 91) |  | 38 (33, 45) |  | 40 (31, 46) |  |
| Age (years) |  |  | <0.001 |  | 0.004 |  | <0.001 |  | 0.578 |
| >30 | 127 (42.3) | 7 (3, 9) |  | 80 (77, 87) |  | 33 (28, 43) |  | 45 (33, 53) |  |
| 31-40 | 127 (42.3) | 8 (5, 9) |  | 83 (78, 90) |  | 41 (33, 46) |  | 40 (31.25, 44) |  |
| 41-50 | 32 (10.7) | 8 (7, 9.75) |  | 90 (79.25, 97) |  | 44 (41.25, 48.75) |  | 37.5 (30.5, 45.25) |  |
| >50 | 14 (4.7) | 1 (1, 6.75) |  | 72 (72, 88.5) |  | 31 (33, 39.5) |  | 40 (31.25, 45) |  |
| Marital status |  |  | 0.088 |  | 0.107 |  | <0.001 |  | 0.946 |
| Single | 99 (33.0) | 6 (2, 9) |  | 80 (77, 86) |  | 34 (27, 42) |  | 42 (26, 46) |  |
| Married | 192 (64.0) | 8 (5, 9) |  | 83 (77, 91) |  | 41 (33, 46) |  | 40 (32.75, 44.75) |  |
| Divorced or widowed | 9 (3.0) | 9 (3.5, 9.5) |  | 84 (75, 96) |  | 42 (35.5, 49.5) |  | 37.5 (29, 46) |  |
| Ethnicity |  |  | 0.361 |  | 0.677 |  | 0.894 |  | 0.268 |
| Han | 291 (97.0) | 7 (4, 9) |  | 82 (77, 90) |  | 38 (33, 45) |  | 40.5 (31.75, 46) |  |
| Minority | 9 (3.0) | 6 (1.5, 9) |  | 85 (76, 90.5) |  | 41 (34, 44) |  | 29 (29, 29) |  |
| Religious belief |  |  | 0.200 |  | 0.335 |  | 0.284 |  | 0.043 |
| Buddhism | 17 (5.7) | 8 (7, 9) |  | 81 (74, 91) |  | 44 (33.5, 53.5) |  | 48 (43, 54.5) |  |
| Taoism | 0 | / |  | / |  | / |  | / |  |
| Christianity | 3 (1.0) | 8 (6, 9) |  | 85 (77, 87) |  | 34 (33, 35) |  | / |  |
| Catholicism | 1 (0.3) | 6 (6,6) |  | 91 (91, 91) |  | 38 (38, 38) |  | / |  |
| Islam | 2 (0.7) | 2 (0, 4) |  | 72 (72, 72) |  | 24.5 (11, 38) |  | / |  |
| None | 277 (92.3) | 7 (4, 9) |  | 81 (74, 91) |  |  |  | 40 (31, 45) |  |
| Education level |  |  | <0.001 |  | <0.001 |  | <0.001 |  | 0.677 |
| Bachelor’s degree and above | 197 (65.7) | 8 (6, 9) |  | 84 (78, 93) |  | 41 (33, 47.5) |  | 40 (31, 46) |  |
| High school and vocational | 90 (30.0) | 6 (2, 9) |  | 80 (74.75, 82) |  | 33 (26, 42) |  | 41.5 (28.25, 44) |  |
| Junior high school and below | 13 (4.3) | 1 (1, 7) |  | 72 (72, 81) |  | 33 (33, 40.5) |  |  |  |
| Workplace location |  |  | 0.676 |  | 0.439 |  | 0.030 |  | 0.014 |
| Chongqing | 159 (53.0) | 8 (4, 9) |  | 82 (75, 90) |  | 40 (33, 45) |  | 42 (36.5, 48.5) |  |
| Hebei province | 141 (47.0) | 7 (4, 9) |  | 82 (78, 90) |  | 35 (33, 44) |  | 34 (27, 43) |  |
| Professional title |  |  | <0.001 |  | <0.001 |  | <0.001 |  | 0.072 |
| Senior | 25 (8.3) | 8 (6, 9.5) |  | 91 (79.5, 97) |  | 44 (38.5, 52.5) |  | 31 (29, 41) |  |
| Intermediate | 106 (35.3) | 8 (6, 9) |  | 84 (80, 91.25) |  | 42 (33, 46) |  | 40 (30.5, 48) |  |
| Junior | 123 (41.0) | 7 (3, 9) |  | 80 (76, 87) |  | 33 (28, 43) |  | 44 (39, 53) |  |
| None | 46 (15.3) | 5 (1, 8) |  | 78 (72,85) |  | 34 (33, 44) |  | 40.5 (29.75, 42) |  |
| Experienced the death of a terminally ill patient or relative |  |  | <0.001 |  | 0.014 |  | 0.007 |  | 0.269 |
| Yes | 227 (75.7) | 8 (5, 9) |  | 82 (78, 91) |  | 40 (33, 45) |  | 40.5 (32.75, 46) |  |
| No | 73 (24.3) | 6 (2, 8) |  | 80 (74, 86) |  | 33 (28, 44.5) |  | 27 (25.5, 46.5) |  |
| Provided hospice care services |  |  | 0.011 |  | 0.685 |  | <0.001 |  |  |
| Yes | 47 (15.7) | 8 (6, 9) |  | 82 (75, 93) |  | 44 (38, 54) |  | 40 (31, 46) |  |
| No | 253 (84.3) | 7 (4, 9) |  | 82 (77.5, 90) |  | 35 (33, 44) |  | / |  |
| Willingness to participate in hospice care services |  |  | <0.001 |  | <0.001 |  | 0.009 |  | 0.113 |
| Yes | 227 (75.7) | 8 (5, 9) |  | 84 (79, 91) |  | 40 (33, 46) |  | 41 (32.5, 46) |  |
| No | 73 (24.3) | 5 (1, 8) |  | 78 (72, 82.5) |  | 33 (33, 41) |  | 29 (29, 29) |  |
| Reasons for willingness to participate in hospice care services (n=227) |  |  | 0.077 |  | <0.001 |  | <0.001 |  | 0.536 |
| Superior’s assigned task | 84 (37.0) | 7.5 (3, 9) |  | 80 (78, 89) |  | 33 (26, 44) |  | 39 (29, 44) |  |
| Personal duty | 123 (54.2) | 8 (6, 9) |  | 87 (81, 94) |  | 43 (34, 49) |  | 42.5 (33.75, 47.5) |  |
| Religious belief | 7 (3.1) | 6 (6, 8) |  | 78 (72, 81) |  | 43 (29, 44) |  | 42 (42, 42) |  |
| Charity work | 13 (5.7) | 7 (3.5, 8.5) |  | 79 (72, 90) |  | 37 (33, 47.5) |  | 42 (39, 45) |  |
| Reasons for unwillingness to participate in hospice care services (n=73) |  |  | 0.726 |  | 0.772 |  | 0.229 |  |  |
| High stress | 46 (63.0) | 6 (1.75, 8) |  | 78.5 (72.75, 82.25) |  | 34 (33, 40.25) |  | 29 (29, 29) |  |
| Low salary | 13 (17.8) | 5 (1.5, 7.5) |  | 78 (69,84) |  | 33 (32, 40.5) |  | / |  |
| Perceived lack of value | 8 (11.0) | 3.5 (1, 8.5) |  | 76 (71.25, 81.5) |  | 33 (28, 37) |  | / |  |
| Seeming meaningless | 5 (6.8) | 1 (1, 8.5) |  | 81 (72, 88) |  | 41 (33, 51) |  | / |  |
| Limited career prospects | 1 (1.4) | 9 (9, 9) |  | 72 (72,72) |  | 43 (43, 43) |  | / |  |

**Table S3.** Comparison of the participants according to workplace location (n=300)

| Variable | Workplace location | | P-value |
| --- | --- | --- | --- |
|  | Chongqing | Hebei Province |  |
| N | 159 | 141 |  |
| Gender |  |  | 0.001 |
| Male | 27 (17.0) | 7 (5.0) |  |
| Female | 132 (83.0) | 134 (95.0) |  |
| Age (years) |  |  | 0.086 |
| <30 | 67 (42.1) | 60 (42.6) |  |
| 31-40 | 63 (39.6) | 64 (45.4) |  |
| 41-50 | 17 (10.7) | 15 (10.6) |  |
| >50 | 12 (7.5) | 2 (1.4) |  |
| Marital status |  |  | 0.745 |
| Single | 55 (34.6) | 44 (31.2) |  |
| Married | 100 (62.9) | 92 (65.2) |  |
| Divorced or widowed | 4 (2.5) | 5 (3.5) |  |
| Ethnicity |  |  | 0.130 |
| Han | 152 (95.6) | 139 (98.6) |  |
| Minority | 7 (4.4) | 2 (1.4) |  |
| Religious belief |  |  | 0.393 |
| Buddhism | 10 (6.3) | 7 (5.0) |  |
| Taoism | 0 | 0 |  |
| Christianity | 1 (0.6) | 2 (1.4) |  |
| Catholicism | 0 | 1 (0.7) |  |
| Islam | 0 | 2 (1.4) |  |
| None | 148 (93.1) | 129 (91.5) |  |
| Education level |  |  | 0.001 |
| Bachelor’s degree and above | 92 (57.9) | 105 (74.5) |  |
| High school and vocational | 55 (34.6) | 35 (24.8) |  |
| Junior high school and below | 12 (7.5) | 1 (0.7) |  |
| Professional title |  |  | <0.001 |
| Senior | 11 (6.9) | 14 (9.9) |  |
| Intermediate | 49 (30.8) | 57 (40.4) |  |
| Junior | 59 (37.1) | 64 (45.4) |  |
| None | 40 (25.2) | 6 (4.3) |  |
| Experienced the death of a terminally ill patient or relative |  |  | 0.468 |
| Yes | 123 (77.4) | 104 (73.8) |  |
| No | 36 (22.6) | 37 (26.2) |  |
| Provided hospice care services |  |  | 0.325 |
| Yes | 28 (17.6) | 19 (13.5) |  |
| No | 131 (82.4) | 122 (86.5) |  |
| Willingness to participate in hospice care services |  |  | 0.089 |
| Yes | 114 (71.7) | 113 (80.1) |  |
| No | 45 (28.3) | 28 (19.9) |  |
| Reasons for willingness to participate in hospice care services (n=227) |  |  | 0.137 |
| Superior’s assigned task | 37 (32.5) | 47 (41.6) |  |
| Personal duty | 63 (55.3) | 60 (53.1) |  |
| Religious belief | 6 (5.3) | 1 (0.9) |  |
| Charity work | 8 (7.0) | 5 (4.4) |  |
| Reasons for unwillingness to participate in hospice care services (n=73) |  |  | 0.826 |
| High stress | 27 (60.0) | 19 (67.9) |  |
| Low salary | 8 (17.8) | 5 (17.9) |  |
| Perceived lack of value | 5 (11.1) | 3 (10.7) |  |
| Seeming meaningless | 4 (8.9) | 1 (3.6) |  |
| Limited career prospects | 1 (2.2) | 0 |  |

**Table S4.** Distribution of the knowledge scores

|  | | | | | | | a. Yes (0) | | b. No (1) | | c. Uncertain (0) | | |  | |
| --- | --- | --- | --- | --- | --- | --- | --- | --- | --- | --- | --- | --- | --- | --- | --- |
| 1. Providing hospice care requires emotional detachment. | | | | | | | 201 (67.0) | | 17 (5.7) | | 82 (27.3) | | |  | |
|  | | | | | | | a. Yes (1) | | b. No (0) | | c. Uncertain (0) | | |  | |
| 2. Psychological, social, and spiritual issues are crucial for a palliative care team to provide appropriate counseling and management. | | | | | | | 239 (79.7) | | 10 (3.3) | | 51 (17.0) | | |  | |
|  | | | | | | | a. Yes (1) | | b. No (0) | | c. Uncertain (0) | | |  | |
| 3. The World Health Organization (WHO) recommends the three-step analgesic ladder for pain management. | | | | | | | 220 (73.3) | | 13 (4.3) | | 67 (22.3) | | |  | |
|  | | | | | | | a. Yes (1) | | b. No (0) | | c. Uncertain (0) | | |  | |
| 4. Hospice care teams offer bereavement support to family members after the patient’s passing. | | | | | | | 189 (63.0) | | 26 (8.7) | | 85 (28.3) | | |  | |
|  | | | | | | | a. Yes (0) | | b. No (1) | | c. Uncertain (0) | | |  | |
| 5. Family-centered hospice care aligns with Chinese cultural practices. | | | | | | | 170 (56.7) | | 23 (7.7) | | 107 (35.7) | | |  | |
|  | | | | | | | a. Yes (1) | | b. No (0) | | c. Uncertain (0) | | |  | |
| 6. For children’s bereavement care, children can participate in funerals and even contribute to the preparations. | | | | | | | 167 (55.7) | | 27 (9.0) | | 106 (35.3) | | |  | |
|  | | | | | | | a. Yes (1) | | b. No (0) | | c. Uncertain (0) | | |  | |
| 7. Medications that can cause respiratory depression are suitable for treating severe respiratory distress in the late stages of illness. | | | | | | | 154 (51.3) | | 52 (17.3) | | 94 (31.3) | | |  | |
|  | | | | | | | a. Yes (0) | | b. No (1) | | c. Uncertain (0) | | |  | |
| 8. Applying potassium permanganate at the Shenque acupoint can alleviate ascites symptoms. | | | | | | | 143 (47.7) | | 11 (3.7) | | 146 (48.7) | | |  | |
|  | | | | | | | a. Yes (1) | | b. No (0) | | c. Uncertain (0) | | |  | |
| 9. Fatigue or anxiety can lower the pain threshold. | | | | | | | 137 (45.7) | | 77 (25.7) | | 86 (28.7) | | |  | |
|  | | | | | | | a. Yes (0) | | b. No (1) | | c. Uncertain (0) | | |  | |
| 10. Men typically recover from grief faster than women. | | | | | | | 158 (52.7) | | 37 (12.3) | | 105 (35.0) | | |  | |
|  | | | | | | | a. Yes (1) | | b. No (0) | | c. Uncertain (0) | | |  | |
| 11. Individuals taking opioid medications should also maintain regular gastrointestinal health. | | | | | | | 193 (64.3) | | 9 (3.0) | | 98 (32.7) | | |  | |
|  | | | | | | | a. Yes (1) | | b. No (0) | | c. Uncertain (0) | | |  | |
| 12. Strengthening hospice care facility construction is included in the “Healthy China 2030” plan. | | | | | | | 198 (66.0) | | 10 (3.3) | | 92 (30.7) | | |  | |
|  | | | | | | | a. Yes (0) | | b. No (1) | | c. Uncertain (0) | | |  | |
| 13. Morphine acupuncture injections can be used to relieve pain in late-stage cancer patients. | | | | | | | 200 (66.7) | | 12 (4.0) | | 88 (29.3) | | |  | |
|  | a. Professional interdisciplinary hospice care teams, including family doctors (1) | | b. Family doctors (0) | | c. Multidisciplinary hospital teams led by pain therapists (0) | | | d. Teams collaborating between professional nursing staff and anesthesiologists (0) | | | | e. Professional nursing staff (0) | | | |
| 14. The most authoritative healthcare planning guidelines recommend that hospice care should be provided by the following teams: | 162 (54.0) | | 14 (4.7) | | 46 (15.3) | | | 26 (8.7) | | | | 52 (17.3) | | | |
|  | | a. Relieving physical pain (0) | | b. Entertainment (1) | | c. Expressing emotions (0) | | | | d. Eliciting memories (0) | | | e. Comforting grief (0) | |  |
| 15. The purpose of music therapy does not include: | | 41 (13.7) | | 181 (60.3) | | 18 (6.0) | | | | 48 (16.0) | | | 12 (4.0) | |  |

**Table S5. Distribution of the attitude scores**

| The worsening condition of late-stage cancer patients makes you feel: | | | | | |
| --- | --- | --- | --- | --- | --- |
|  | a. Strongly agree (1) | b. Partially agree (2) | c. Neutral (3) | d. Partially disagree (4) | e. Strongly disagree (5) |
| 1. Uncomfortable caring for late-stage cancer patients. | 95 (31.7) | 56 (18.7) | 91 (30.3) | 23 (7.7) | 35 (11.7) |
|  | a. Strongly agree (1) | b. Partially agree (2) | c. Neutral (3) | d. Partially disagree (4) | e. Strongly disagree (5) |
| 2. Hopeless about a cure. | 103 (34.3) | 62 (20.7) | 91 (30.3) | 26 (8.7) | 18 (6.0) |
|  | a. Strongly agree (1) | b. Partially agree (2) | c. Neutral (3) | d. Partially disagree (4) | e. Strongly disagree (5) |
| 3. Unable to face the dying process and suffering with ease. | 103 (34.3) | 70 (23.3) | 87 (29.0) | 23 (7.7) | 17 (5.7) |
|  | a. Strongly agree (1) | b. Partially agree (2) | c. Neutral (3) | d. Partially disagree (4) | e. Strongly disagree (5) |
| 4. Powerless. | 111 (37.0) | 66 (22.0) | 88 (29.3) | 19 (6.3) | 16 (5.3) |
|  | a. Strongly agree (1) | b. Partially agree (2) | c. Neutral (3) | d. Partially disagree (4) | e. Strongly disagree (5) |
| 5. Guilty when the patient dies. | 74 (24.7) | 75 (25.0) | 93 (31.0) | 26 (8.7) | 32 (10.7) |
| Do you believe that improving the quality of life can: |  |  |  |  |  |
|  | a. Strongly agree (5) | b. Partially agree (4) | c. Neutral (3) | d. Partially disagree (2) | e. Strongly disagree (1) |
| 6. Promote quality of life and maintain dignity. | 192 (64.0) | 40 (13.3) | 57 (19.0) | 8 (2.7) | 3 (1.0) |
|  | a. Strongly agree (5) | b. Partially agree (4) | c. Neutral (3) | d. Partially disagree (2) | e. Strongly disagree (1) |
| 7. Enable a peaceful and fulfilling death. | 178 (59.3) | 56 (18.7) | 54 (18.0) | 6 (2.0) | 6 (2.0) |
|  | a. Strongly agree (5) | b. Partially agree (4) | c. Neutral (3) | d. Partially disagree (2) | e. Strongly disagree (1) |
| 8. Provide care and companionship from the medical team. | 185 (61.7) | 48 (16.0) | 59 (19.7) | 6 (2.0) | 2 (0.7) |
|  | a. Strongly agree (5) | b. Partially agree (4) | c. Neutral (3) | d. Partially disagree (2) | e. Strongly disagree (1) |
| 9. Offer emotional support. | 194 (64.7) | 50 (16.7) | 50 (16.7) | 4 (1.3) | 2 (0.7) |
|  | a. Strongly agree (5) | b. Partially agree (4) | c. Neutral (3) | d. Partially disagree (2) | e. Strongly disagree (1) |
| 10. Receive support from the family. | 194 (64.7) | 50 (16.7) | 49 (16.3) | 4 (1.3) | 3 (1.0) |
| Do you think that better preparing for death involves: |  |  |  |  |  |
|  | a. Strongly agree (5) | b. Partially agree (4) | c. Neutral (3) | d. Partially disagree (2) | e. Strongly disagree (1) |
| 11. Respecting the patient’s religious beliefs and funeral rituals. | 193 (64.3) | 48 (16.0) | 54 (18.0) | 3 (1.0) | 2 (0.7) |
|  | a. Strongly agree (5) | b. Partially agree (4) | c. Neutral (3) | d. Partially disagree (2) | e. Strongly disagree (1) |
| 12. Assisting the patient in peacefully passing away at home. | 171 (57.0) | 54 (18.0) | 63 (21.0) | 6 (2.0) | 6 (2.0) |
|  | a. Strongly agree (5) | b. Partially agree (4) | c. Neutral (3) | d. Partially disagree (2) | e. Strongly disagree (1) |
| 13. Communicating better with terminally ill patients. | 180 (60.0) | 58 (19.3) | 53 (17.7) | 6 (2.0) | 3 (1.0) |
|  | a. Strongly agree (5) | b. Partially agree (4) | c. Neutral (3) | d. Partially disagree (2) | e. Strongly disagree (1) |
| 14. Helping healthcare providers better care for patients. | 190 (63.3) | 52 (17.3) | 48 (16.0) | 6 (2.0) | 4 (1.3) |
|  | a. Strongly agree (5) | b. Partially agree (4) | c. Neutral (3) | d. Partially disagree (2) | e. Strongly disagree (1) |
| 15. Avoiding euthanasia concepts. | 115 (38.3) | 52 (17.3) | 100 (33.3) | 20 (16.7) | 13 (4.3) |
| Obstacles to providing hospice care include： |  |  |  |  |  |
|  | a. Strongly agree (1) | b. Partially agree (2) | c. Neutral (3) | d. Partially disagree (4) | e. Strongly disagree (5) |
| 16. Shortening the patient’s life, similar to euthanasia. | 90 (30.0) | 47 (15.7) | 81 (27.0) | 35 (11.7) | 47 (15.7) |
|  | a. Strongly agree (1) | b. Partially agree (2) | c. Neutral (3) | d. Partially disagree (4) | e. Strongly disagree (5) |
| 17. Not actively treating physical symptoms. | 85 (28.3) | 53 (17.7) | 86 (28.7) | 30 (10.0) | 46 (15.3) |
|  | a. Strongly agree (1) | b. Partially agree (2) | c. Neutral (3) | d. Partially disagree (4) | e. Strongly disagree (5) |
| 18. Making the patient feel hopeless. | 81 (27.0) | 39 (13.0) | 84 (28.0) | 41 (13.7) | 55 (18.3) |
|  | a. Strongly agree (1) | b. Partially agree (2) | c. Neutral (3) | d. Partially disagree (4) | e. Strongly disagree (5) |
| 19. Late-stage patients have many challenging symptoms. | 129 (43.0) | 67 (22.3) | 76 (25.3) | 18 (6.0) | 10 (3.3) |
|  | a. Strongly agree (1) | b. Partially agree (2) | c. Neutral (3) | d. Partially disagree (4) | e. Strongly disagree (5) |
| 20. Losing enthusiasm for providing long-term hospice care. | 94 (31.3) | 62 (20.7) | 96 (32.0) | 27 (9.0) | 21 (7.0) |
| Providing hospice care: |  |  |  |  |  |
|  | a. Strongly agree (5) | b. Partially agree (4) | c. Neutral (3) | d. Partially disagree (2) | e. Strongly disagree (1) |
| 21. Is meaningful. | 173 (57.7) | 62 (20.7) | 60 (2.0) | 3 (1.0) | 2 (0.7) |
|  | a. Strongly agree (5) | b. Partially agree (4) | c. Neutral (3) | d. Partially disagree (2) | e. Strongly disagree (1) |
| 22. Has been influenced by my family’s experience of death. | 80 (26.7) | 53 (17.7) | 101 (33.7) | 37 (12.3) | 29 (9.7) |
|  | a. Strongly agree (5) | b. Partially agree (4) | c. Neutral (3) | d. Partially disagree (2) | e. Strongly disagree (1) |
| 23. Is part of the responsibilities of healthcare professionals. | 144 (48.0) | 64 (21.3) | 85 (28.3) | 5 (1.7) | 2 (0.7) |
|  | a. Strongly agree (5) | b. Partially agree (4) | c. Neutral (3) | d. Partially disagree (2) | e. Strongly disagree (1) |
| 24. Is encouraged by department leaders, colleagues, friends, and family’s recognition and support. | 137 (45.7) | 68 (22.7) | 87 (29.0) | 5 (1.7) | 3 (1.0) |

**Table S6. Distribution of the practice scores**

| How confident are you personally in providing hospice care? |  |  |  |  |  |
| --- | --- | --- | --- | --- | --- |
|  | a. Very confident (5) | b. Confident (4) | c. Neutral (3) | d. Unconfident (2) | e. Very unconfident (1) |
| 1. Alleviating the pain and discomfort of terminally ill patients. | 66 (22.0) | 82 (27.3) | 107 (35.7) | 38 (12.7) | 7 (2.3) |
|  | a. Very confident (5) | b. Confident (4) | c. Neutral (3) | d. Unconfident (2) | e. Very unconfident (1) |
| 2. Conducting pain assessments on patients. | 61 (20.3) | 106 (35.3) | 106 (35.3) | 21 (7.0) | 6 (2.0) |
|  | a. Very confident (5) | b. Confident (4) | c. Neutral (3) | d. Unconfident (2) | e. Very unconfident (1) |
| 3. Reducing unnecessary treatment costs. | 54 (18.0) | 76 (25.3) | 115 (38.3) | 45 (15.0) | 10 (3.3) |
|  | a. Very confident (5) | b. Confident (4) | c. Neutral (3) | d. Unconfident (2) | e. Very unconfident (1) |
| 4. Meeting the physical and psychological needs of terminally ill patients. | 63 (21.0) | 87 (29.0) | 98 (32.7) | 39 (13.0) | 13 (4.3) |
|  | a. Very confident (5) | b. Confident (4) | c. Neutral (3) | d. Unconfident (2) | e. Very unconfident (1) |
| 5. Explaining the expected dying process to patients’ families. | 61 (20.3) | 75 (25.0) | 115 (38.3) | 40 (13.3) | 9 (3.0) |
|  | a. Very confident (5) | b. Confident (4) | c. Neutral (3) | d. Unconfident (2) | e. Very unconfident (1) |
| 6. Informing families about what they can do to provide meaningful service to the patient. | 70 (23.3) | 92 (30.7) | 94 (31.3) | 38 (12.7) | 6 (2.0) |
|  | a. Very confident (5) | b. Confident (4) | c. Neutral (3) | d. Unconfident (2) | e. Very unconfident (1) |
| 7. Understanding the wishes and sorrows of families to assist them. | 65 (21.7) | 85 (28.3) | 103 (34.3) | 38 (12.7) | 9 (3.0) |
|  | a. Very confident (5) | b. Confident (4) | c. Neutral (3) | d. Unconfident (2) | e. Very unconfident (1) |
| 8. Establishing a good relationship between healthcare providers and families. | 66 (22.0) | 108 (36.0) | 95 (31.7) | 24 (8.0) | 7 (2.3) |
|  | a. Very confident (5) | b. Confident (4) | c. Neutral (3) | d. Unconfident (2) | e. Very unconfident (1) |
| 9. Coordinating resources in medical, social, psychological, and mental care. | 66 (22.0) | 81 (27.0) | 102 (34.0) | 44 (14.7) | 7 (2.3) |
|  | a. Very confident (5) | b. Confident (4) | c. Neutral (3) | d. Unconfident (2) | e. Very unconfident (1) |
| 10. Helping grieving families better navigate difficult times. | 59 (19.7) | 83 (27.7) | 104 (34.7) | 46 (15.3) | 8 (2.7) |
|  | a. Very confident (5) | b. Confident (4) | c. Neutral (3) | d. Unconfident (2) | e. Very unconfident (1) |
| 11. Guiding families in making post-death and funeral preparations. | 59 (19.7) | 78 (26.0) | 108 (36.0) | 49 (16.3) | 6 (2.0) |
| If you have experience in hospice care, please provide details of your actual job responsibilities (you may skip this section if you have no experience). Have experience in hospice care: N=47 | | | | | |
|  | a. Never (1) | b. Rarely (2) | c. Sometimes (3) | d. Often (4) | e. Always (5) |
| 1. Alleviating the pain and discomfort of terminally ill patients. | 2 (4.3) | 2 (4.3) | 17 (36.2) | 16 (34.0) | 10 (21.3) |
|  | a. Never (1) | b. Rarely (2) | c. Sometimes (3) | d. Often (4) | e. Always (5) |
| 2. Conducting pain assessments on patients. | 4 (8.5) | 6 (12.8) | 8 (17.0) | 15 (31.9) | 14 (29.8) |
|  | a. Never (1) | b. Rarely (2) | c. Sometimes (3) | d. Often (4) | e. Always (5) |
| 3. Reducing unnecessary treatment costs. | 4 (8.5) | 4 (8.5) | 11 (23.4) | 20 (42.6) | 8 (17.0) |
|  | a. Never (1) | b. Rarely (2) | c. Sometimes (3) | d. Often (4) | e. Always (5) |
| 4. Meeting the physical and psychological needs of terminally ill patients. | 4 (8.5) | 3 (6.4) | 8 (17.0) | 19 (40.4) | 13 (27.7) |
|  | a. Never (1) | b. Rarely (2) | c. Sometimes (3) | d. Often (4) | e. Always (5) |
| 5. Explaining the expected dying process to patients’ families. | 3 (6.4) | 7 (14.9) | 12 (25.5) | 17 (36.2) | 8 (17.0) |
|  | a. Never (1) | b. Rarely (2) | c. Sometimes (3) | d. Often (4) | e. Always (5) |
| 6. Informing families about what they can do to provide meaningful service to the patient. | 2 (4.3) | 6 (12.8) | 9 (19.1) | 19 (40.4) | 11 (23.4) |
|  | a. Never (1) | b. Rarely (2) | c. Sometimes (3) | d. Often (4) | e. Always (5) |
| 7. Understanding the wishes and sorrows of families to assist them. | 2 (4.3) | 6 (12.8) | 12 (25.5) | 16 (34.0) | 11 (23.4) |
|  | a. Never (1) | b. Rarely (2) | c. Sometimes (3) | d. Often (4) | e. Always (5) |
| 8. Establishing a good relationship between healthcare providers and families. | 2 (4.3) | 8 (17.0) | 7 (14.9) | 17 (36.2) | 13 (27.7) |
|  | a. Never (1) | b. Rarely (2) | c. Sometimes (3) | d. Often (4) | e. Always (5) |
| 9. Coordinating resources in medical, social, psychological, and mental care. | 1 (2.1) | 17 (36.2) | 8 (17.0) | 11 (23.4) | 10 (21.3) |
|  | a. Never (1) | b. Rarely (2) | c. Sometimes (3) | d. Often (4) | e. Always (5) |
| 10. Assisting grieving families in better coping with difficult times. | 1 (2.1) | 7 (14.9) | 13 (27.7) | 17 (36.2) | 9 (19.1) |
|  | a. Never (1) | b. Rarely (2) | c. Sometimes (3) | d. Often (4) | e. Always (5) |
| 11. Guiding families in making post-death and funeral preparations. | 1 (2.1) | 9 (19.1) | 13 (27.7) | 14 (29.8) | 10 (21.3) |

**Table S7. Distribution of the practice scores in Chongqing**

| How confident are you personally in providing hospice care? |  |  |  |  |  |
| --- | --- | --- | --- | --- | --- |
|  | a. Very confident (5) | b. Confident (4) | c. Neutral (3) | d. Unconfident (2) | e. Very unconfident (1) |
| 1. Alleviating the pain and discomfort of terminally ill patients. | 34 (21.4) | 52 (32.7) | 55 (34.6) | 16 (10.1) | 2 (1.3) |
|  | a. Very confident (5) | b. Confident (4) | c. Neutral (3) | d. Unconfident (2) | e. Very unconfident (1) |
| 2. Conducting pain assessments on patients. | 32 (20.1) | 64 (40.3) | 54 (34.0) | 8 (5.0) | 1 (0.6) |
|  | a. Very confident (5) | b. Confident (4) | c. Neutral (3) | d. Unconfident (2) | e. Very unconfident (1) |
| 3. Reducing unnecessary treatment costs. | 30 (18.9) | 41 (25.8) | 65 (40.9) | 20 (12.6) | 3 (1.9) |
|  | a. Very confident (5) | b. Confident (4) | c. Neutral (3) | d. Unconfident (2) | e. Very unconfident (1) |
| 4. Meeting the physical and psychological needs of terminally ill patients. | 37 (23.3) | 50 (31.4) | 48 (30.2) | 19 (11.9) | 5 (3.1) |
|  | a. Very confident (5) | b. Confident (4) | c. Neutral (3) | d. Unconfident (2) | e. Very unconfident (1) |
| 5. Explaining the expected dying process to patients’ families. | 37 (23.3) | 43 (27.0) | 61 (38.4) | 16 (10.1) | 2 (1.3) |
|  | a. Very confident (5) | b. Confident (4) | c. Neutral (3) | d. Unconfident (2) | e. Very unconfident (1) |
| 6. Informing families about what they can do to provide meaningful service to the patient. | 41 (25.8) | 53 (33.3) | 51 (32.1) | 14 (8.8) | 0 |
|  | a. Very confident (5) | b. Confident (4) | c. Neutral (3) | d. Unconfident (2) | e. Very unconfident (1) |
| 7. Understanding the wishes and sorrows of families to assist them. | 39 (24.5) | 50 (31.4) | 53 (33.3) | 15 (9.4) | 2 (1.3) |
|  | a. Very confident (5) | b. Confident (4) | c. Neutral (3) | d. Unconfident (2) | e. Very unconfident (1) |
| 8. Establishing a good relationship between healthcare providers and families. | 39 (24.5) | 63 (39.6) | 49 (30.8) | 6 (3.8) | 2 (1.3) |
|  | a. Very confident (5) | b. Confident (4) | c. Neutral (3) | d. Unconfident (2) | e. Very unconfident (1) |
| 9. Coordinating resources in medical, social, psychological, and mental care. | 38 (23.9) | 46 (28.9) | 54 (34.0) | 19 (11.9) | 2 (1.3) |
|  | a. Very confident (5) | b. Confident (4) | c. Neutral (3) | d. Unconfident (2) | e. Very unconfident (1) |
| 10. Helping grieving families better navigate difficult times. | 37 (23.3) | 47 (29.6) | 55 (34.6) | 19 (11.9) | 1 (0.6) |
|  | a. Very confident (5) | b. Confident (4) | c. Neutral (3) | d. Unconfident (2) | e. Very unconfident (1) |
| 11. Guiding families in making post-death and funeral preparations. | 36 (22.6) | 44 (27.7) | 58 (36.5) | 20 (12.6) | 1 (0.6) |
| If you have experience in hospice care, please provide details of your actual job responsibilities (you may skip this section if you have no experience). Have experience in hospice care: N=28 | | | | | |
|  | a. Never (1) | b. Rarely (2) | c. Sometimes (3) | d. Often (4) | e. Always (5) |
| 1. Alleviating the pain and discomfort of terminally ill patients. | 0 | 1 (3.6) | 9 (32.1) | 11 (39.3) | 7 (25.0) |
|  | a. Never (1) | b. Rarely (2) | c. Sometimes (3) | d. Often (4) | e. Always (5) |
| 2. Conducting pain assessments on patients. | 0 | 5 (17.9) | 5 (17.9) | 7 (25.0) | 11 (39.3) |
|  | a. Never (1) | b. Rarely (2) | c. Sometimes (3) | d. Often (4) | e. Always (5) |
| 3. Reducing unnecessary treatment costs. | 1 (3.6) | 3 (10.7) | 5 (17.9) | 15 (53.6) | 4 (14.3) |
|  | a. Never (1) | b. Rarely (2) | c. Sometimes (3) | d. Often (4) | e. Always (5) |
| 4. Meeting the physical and psychological needs of terminally ill patients. | 2 (7.1) | 1 (3.6) | 4 (14.3) | 9 (32.1) | 12 (42.9) |
|  | a. Never (1) | b. Rarely (2) | c. Sometimes (3) | d. Often (4) | e. Always (5) |
| 5. Explaining the expected dying process to patients’ families. | 1 (3.6) | 1 (3.6) | 8 (28.6) | 11 (39.3) | 7 (25.0) |
|  | a. Never (1) | b. Rarely (2) | c. Sometimes (3) | d. Often (4) | e. Always (5) |
| 6. Informing families about what they can do to provide meaningful service to the patient. | 0 | 2 (7.1) | 6 (21.4) | 11 (39.3) | 9 (32.1) |
|  | a. Never (1) | b. Rarely (2) | c. Sometimes (3) | d. Often (4) | e. Always (5) |
| 7. Understanding the wishes and sorrows of families to assist them. | 0 | 3 (10.7) | 5 (17.9) | 11 (39.3) | 9 (32.1) |
|  | a. Never (1) | b. Rarely (2) | c. Sometimes (3) | d. Often (4) | e. Always (5) |
| 8. Establishing a good relationship between healthcare providers and families. | 0 | 4 (14.3) | 4 (14.3) | 9 (32.1) | 11 (39.3) |
|  | a. Never (1) | b. Rarely (2) | c. Sometimes (3) | d. Often (4) | e. Always (5) |
| 9. Coordinating resources in medical, social, psychological, and mental care. | 0 | 7 (25.0) | 5 (17.9) | 7 (25.0) | 9 (32.1) |
|  | a. Never (1) | b. Rarely (2) | c. Sometimes (3) | d. Often (4) | e. Always (5) |
| 10. Helping grieving families better navigate difficult times. | 0 | 3 (10.7) | 7 (25.0) | 10 (35.7) | 8 (28.6) |
|  | a. Never (1) | b. Rarely (2) | c. Sometimes (3) | d. Often (4) | e. Always (5) |
| 11. Guiding families in making post-death and funeral preparations. | 0 | 3 (10.7) | 9 (32.1) | 9 (32.1) | 7 (25.0) |

**Table S8. Distribution of the practice scores in Hebei**

| How confident are you personally in providing hospice care? |  |  |  |  |  |
| --- | --- | --- | --- | --- | --- |
|  | a. Very confident (5) | b. Confident (4) | c. Neutral (3) | d. Unconfident (2) | e. Very unconfident (1) |
| 1. Alleviating the pain and discomfort of terminally ill patients. | 32 (22.7) | 30 (21.3) | 52 (36.9) | 22 (15.6) | 5 (3.5) |
|  | a. Very confident (5) | b. Confident (4) | c. Neutral (3) | d. Unconfident (2) | e. Very unconfident (1) |
| 2. Conducting pain assessments on patients. | 29 (20.6) | 42 (29.8) | 52 (36.9) | 13 (9.2) | 5 (3.5) |
|  | a. Very confident (5) | b. Confident (4) | c. Neutral (3) | d. Unconfident (2) | e. Very unconfident (1) |
| 3. Reducing unnecessary treatment costs. | 24 (17.0) | 35 (24.8) | 50 (35.5) | 25 (17.7) | 7 (5.0) |
|  | a. Very confident (5) | b. Confident (4) | c. Neutral (3) | d. Unconfident (2) | e. Very unconfident (1) |
| 4. Meeting the physical and psychological needs of terminally ill patients. | 26 (18.4) | 37 (26.2) | 50 (35.5) | 20 (14.2) | 8 (5.7) |
|  | a. Very confident (5) | b. Confident (4) | c. Neutral (3) | d. Unconfident (2) | e. Very unconfident (1) |
| 5. Explaining the expected dying process to patients’ families. | 24 (17.0) | 32 (22.7) | 54 (38.3) | 24 (17.0) | 7 (5.0) |
|  | a. Very confident (5) | b. Confident (4) | c. Neutral (3) | d. Unconfident (2) | e. Very unconfident (1) |
| 6. Informing families about what they can do to provide meaningful service to the patient. | 29 (20.6) | 39 (27.7) | 43 (30.5) | 24 (17.0) | 6 (4.3) |
|  | a. Very confident (5) | b. Confident (4) | c. Neutral (3) | d. Unconfident (2) | e. Very unconfident (1) |
| 7. Understanding the wishes and sorrows of families to assist them. | 26 (18.4) | 35 (24.8) | 50 (35.5) | 23 (16.3) | 7 (5.0) |
|  | a. Very confident (5) | b. Confident (4) | c. Neutral (3) | d. Unconfident (2) | e. Very unconfident (1) |
| 8. Establishing a good relationship between healthcare providers and families. | 27 (19.1) | 45 (31.9) | 46 (32.6) | 18 (12.8) | 5 (3.5) |
|  | a. Very confident (5) | b. Confident (4) | c. Neutral (3) | d. Unconfident (2) | e. Very unconfident (1) |
| 9. Coordinating resources in medical, social, psychological, and mental care. | 28 (19.9) | 35 (24.8) | 48 (34.0) | 25 (17.7) | 7 (5.0) |
|  | a. Very confident (5) | b. Confident (4) | c. Neutral (3) | d. Unconfident (2) | e. Very unconfident (1) |
| 10. Helping grieving families better navigate difficult times. | 22 (15.6) | 36 (25.5) | 49 (34.8) | 27 (19.1) | 7 (5.0) |
|  | a. Very confident (5) | b. Confident (4) | c. Neutral (3) | d. Unconfident (2) | e. Very unconfident (1) |
| 11. Guiding families in making post-death and funeral preparations. | 23 (16.3) | 34 (24.1) | 50 (35.5) | 29 (20.6) | 5 (3.5) |
| If you have experience in hospice care, please provide details of your actual job responsibilities (you may skip this section if you have no experience). Have experience in hospice care: N=19 | | | | | |
|  | a. Never (1) | b. Rarely (2) | c. Sometimes (3) | d. Often (4) | e. Always (5) |
| 1. Alleviating the pain and discomfort of terminally ill patients. | 2 (10.5) | 1 (5.3) | 8 (42.1) | 5 (26.3) | 3 (15.8) |
|  | a. Never (1) | b. Rarely (2) | c. Sometimes (3) | d. Often (4) | e. Always (5) |
| 2. Conducting pain assessments on patients. | 4 (21.1) | 1 (5.3) | 3 (15.8) | 8 (42.1) | 3 (15.8) |
|  | a. Never (1) | b. Rarely (2) | c. Sometimes (3) | d. Often (4) | e. Always (5) |
| 3. Reducing unnecessary treatment costs. | 3 (15.8) | 1 (5.3) | 6 (31.6) | 5 (26.3) | 4 (21.1) |
|  | a. Never (1) | b. Rarely (2) | c. Sometimes (3) | d. Often (4) | e. Always (5) |
| 4. Meeting the physical and psychological needs of terminally ill patients. | 2 (10.5) | 2 (10.5) | 4 (21.1) | 10 (52.6) | 1 (5.3) |
|  | a. Never (1) | b. Rarely (2) | c. Sometimes (3) | d. Often (4) | e. Always (5) |
| 5. Explaining the expected dying process to patients’ families. | 2 (10.5) | 6 (31.6) | 4 (21.1) | 6 (31.6) | 1 (5.3) |
|  | a. Never (1) | b. Rarely (2) | c. Sometimes (3) | d. Often (4) | e. Always (5) |
| 6. Informing families about what they can do to provide meaningful service to the patient. | 2 (10.5) | 4 (21.1) | 3 (15.8) | 8 (42.1) | 2 (10.5) |
|  | a. Never (1) | b. Rarely (2) | c. Sometimes (3) | d. Often (4) | e. Always (5) |
| 7. Understanding the wishes and sorrows of families to assist them. | 2 (10.5) | 3 (15.8) | 7 (36.8) | 5 (26.3) | 2 (10.5) |
|  | a. Never (1) | b. Rarely (2) | c. Sometimes (3) | d. Often (4) | e. Always (5) |
| 8. Establishing a good relationship between healthcare providers and families. | 2 (10.5) | 4 (21.1) | 3 (15.8) | 8 (42.1) | 2 (10.5) |
|  | a. Never (1) | b. Rarely (2) | c. Sometimes (3) | d. Often (4) | e. Always (5) |
| 9. Coordinating resources in medical, social, psychological, and mental care. | 1 (5.3) | 10 (52.6) | 3 (15.8) | 4 (21.1) | 1 (5.3) |
|  | a. Never (1) | b. Rarely (2) | c. Sometimes (3) | d. Often (4) | e. Always (5) |
| 10. Helping grieving families better navigate difficult times. | 1 (5.3) | 4 (21.1) | 6 (31.6) | 7 (36.8) | 1 (5.3) |
|  | a. Never (1) | b. Rarely (2) | c. Sometimes (3) | d. Often (4) | e. Always (5) |
| 11. Guiding families in making post-death and funeral preparations. | 1 (5.3) | 6 (31.6) | 4 (21.1) | 5 (26.3) | 1. (15.8) |

**Table S9. Correlation analysis in Chongqing**

| n=159 | | Knowledge | | Attitude | | Confidence in practice | | |
| --- | --- | --- | --- | --- | --- | --- | --- | --- |
| Knowledge | | 1.000 | | / | | / | | |
| Attitude | | 0.321 (P<0.001) | | 1.000 | | / | | |
| Confidence in practice | | 0.369 (P<0.001) | | 0.573 (P<0.001) | | 1.000 | | |
| n=28 | Knowledge | | Attitude | | Confidence in practice | | Actual practice |  |
| Knowledge | 1.000 | | / | | / | | / |  |
| Attitude | 0.330 (P=0.086) | | 1.000 | | / | | / |  |
| Confidence in practice | 0.522 (P=0.004) | | 0.467 (P=0.012) | | 1.000 | | / |  |
| Actual practice | 0.151 (P=0.443) | | 0.481 (P=0.010) | | 0.414 (P=0.029) | | 1.000 |  |

**Table S10. Correlation analysis in Hebei**

| n=141 | | Knowledge | Attitude | Confidence in practice | |  |
| --- | --- | --- | --- | --- | --- | --- |
| Knowledge | | 1.000 | / | / | |  |
| Attitude | | 0.371 (P<0.001) | 1.000 | / | |  |
| Confidence in practice | | 0.368 (P<0.001) | 0.465 (P<0.001) | 1.000 | |  |
| n=19 | Knowledge | | Attitude | Confidence in practice | Actual practice | |
| Knowledge | 1.000 | | / | / | / | |
| Attitude | 0.535 (P=0.018) | | 1.000 | / | / | |
| Confidence in practice | 0.451 (P=0.053) | | 0.642 (P=0.003) | 1.000 | / | |
| Actual practice | 0.432 (P=0.065) | | 0.358 (P=0.132) | 0.202 (P=0.406) | 1.000 | |

**Table S11.** Univariable and multivariable regression analyses of practice in Chongqing

| Cut-off value: ≥38/<38 | No. | Univariable | | Multivariable (enter method) | |
| --- | --- | --- | --- | --- | --- |
|  |  | OR (95%CI) | P | OR (95%CI) | P |
| Gender |  |  |  |  |  |
| Male | 14/27 | 0.746 (0.325, 1.711) | 0.489 |  |  |
| Female | 78/132 | ref. |  |  |  |
| Age (years) (comparison method: difference) |  |  |  |  |  |
| <30 | 34/67 | ref. |  |  |  |
| 31-40 | 42/63 | 1.914 (0.955, 3.947) | 0.067 |  |  |
| 41-50 | 13/17 | 2.264 (0.699, 7.335) | 0.173 |  |  |
| >50 | 3/12 | 0.177 (0.045, 0.703) | 0.014 |  |  |
| Marital status |  |  |  |  |  |
| Single | 30/55 | ref. |  |  |  |
| Married | 59/100 | 1.199 (0.618, 2.329) | 0.592 |  |  |
| Divorced or widowed | 3/4 | 2.500 (0.245,25.556) | 0.440 |  |  |
| Ethnicity |  |  |  |  |  |
| Han | 87/152 | ref. |  |  |  |
| Minority | 5/7 | 1.868 (0.351, 9.932) | 0.464 |  |  |
| Religious belief |  |  |  |  |  |
| Yes | 7/11 | 1.297 (0.364, 4.623) | 0.688 |  |  |
| No | 85/148 | ref. |  |  |  |
| Education level |  |  |  |  |  |
| Bachelor’s degree and above | 63/92 | ref. |  | ref. |  |
| High school and vocational | 26/55 | 0.413 (0.207, 0.821) | 0.012 | 1.132 (0.457, 2.807) | 0.789 |
| Junior high school and below | 3/12 | 0.153 (0.039, 0.609) | 0.008 | 0.908 (0.185, 4.464) | 0.906 |
| Professional title |  |  |  |  |  |
| Senior | 9/11 | ref. |  |  |  |
| Intermediate | 34/49 | 0.504 (0.097,2.618) | 0.415 |  |  |
| Junior | 29/59 | 0.215 (0.043, 1.080) | 0.062 |  |  |
| None | 20/40 | 0.222 (0.043, 1.160) | 0.074 |  |  |
| Experienced the death of a terminally ill patient or relative |  |  |  |  |  |
| Yes | 78/123 | ref. |  | ref. |  |
| No | 14/36 | 0.367 (0.171, 0.788) | 0.010 | 0.465 (0.170, 1.273) | 0.136 |
| Provided hospice care services |  |  |  |  |  |
| Yes | 23/28 | ref. |  | ref. |  |
| No | 69/131 | 0.242 (0.087, 0.675) | 0.007 | 0.266 (0.079, 0.890) | 0.032 |
| Willingness to participate in hospice care services |  |  |  |  |  |
| Yes | 76/114 | ref. |  | ref. |  |
| No | 16/45 | 0.276 (0.134, 0.569) | <0.001 | 0.806 (0.321, 2.026) | 0.647 |
| Knowledge score |  |  |  |  |  |
| <7 | 22/58 | ref. |  | ref. |  |
| >7 | 70/101 | 3.695 (1.875, 7.281) | <0.001 | 1.969 (0.857, 4.521) | 0.110 |
| Attitude score |  |  |  |  |  |
| <82 | 25/78 | ref. |  | ref. |  |
| >82 | 67/81 | 10.146 (4.807, 21.412) | <0.001 | 9.305 (3.850, 22.488) | <0.001 |

**Table S12.** Univariable and multivariable regression analyses of practice in Hebei

| Cut-off value: ≥38/<38 | No. | Univariable | | Multivariable (enter method) | |
| --- | --- | --- | --- | --- | --- |
|  |  | OR (95%CI) | P | OR (95%CI) | P |
| Gender |  |  |  |  |  |
| Male | 2/7 | 0.479 (0.090, 2.555) | 0.389 |  |  |
| Female | 61/134 | ref. |  |  |  |
| Age (years) (comparison method: difference) |  |  |  |  |  |
| <30 | 14/60 | ref. |  |  |  |
| 31-40 | 34/64 | 3.724 (1.717, 8.074) | <0.001 |  |  |
| 41-50 | 14/15 | 23.838 (3.022, 188.029) | 0.003 |  |  |
| >50 | 1/2 | 0.592 (0.034, 10.380) | 0.720 |  |  |
| Marital status |  |  |  |  |  |
| Single | 8/44 | ref. |  | ref. |  |
| Married | 52/92 | 5.850 (2.451, 13.963) | <0.001 | 2.773 (1.053, 7.306) | 0.039 |
| Divorced or widowed | 3/5 | 6.750 (0.964, 47.269) | 0.054 | 2.076 (0.227, 18.991) | 0.518 |
| Ethnicity |  |  |  |  |  |
| Han | 63/139 | / |  |  |  |
| Minority | 0/2 | / |  |  |  |
| Religious belief |  |  |  |  |  |
| Yes | 6/12 | 1.263 (0.387, 4.126) | 0.699 |  |  |
| No | 57/129 | ref. |  |  |  |
| Education level |  |  |  |  |  |
| Bachelor’s degree and above | 56/105 | ref. |  |  |  |
| High school and vocational | 6/35 | 0.181 (0.069, 0.472) | <0.001 |  |  |
| Junior high school and below | 1/1 | / |  |  |  |
| Professional title |  |  |  |  |  |
| Senior | 11/14 | ref. |  |  |  |
| Intermediate | 33/57 | 0.375 (0.094, 1.492) | 0.164 |  |  |
| Junior | 17/64 | 0.099 (0.025, 0.397) | 0.001 |  |  |
| None | 2/6 | 0.136 (0.016, 1.140) | 0.066 |  |  |
| Experienced the death of a terminally ill patient or relative |  |  |  |  |  |
| Yes | 50/104 | ref. |  |  |  |
| No | 13/37 | 0.585 (0.269, 1.272) | 0.176 |  |  |
| Provided hospice care services |  |  |  |  |  |
| Yes | 15/19 | ref. |  | ref. |  |
| No | 48/122 | 0.173 (0.054, 0.552) | 0.003 | 0.132 (0.034, 0.520) | 0.004 |
| Willingness to participate in hospice care services |  |  |  |  |  |
| Yes | 53/113 | ref. |  |  |  |
| No | 10/28 | 0.629 (0.267, 1.481) | 0.289 |  |  |
| Knowledge score |  |  |  |  |  |
| <7 | 19/63 | ref. |  | ref. |  |
| >7 | 44/78 | 2.997 (1.488,6.034) | 0.002 | 1.830 (0.802,4.172) | 0.151 |
| Attitude score |  |  |  |  |  |
| <82 | 16/68 | ref. |  | ref. |  |
| >82 | 47/73 | 5.875 (2.811, 12.279) | <0.001 | 5.401 (2.269, 12.857) | <0.001 |
